# One syndrome, many diseases: toward precision pharmacotherapy in acute respiratory distress syndrome

**DOI:** 10.3389/fphar.2026.1924674

**Published:** 2026-08-24

**Authors:** Raffaele Merola, Denise Battaglini, Patricia R. M. Rocco

**Affiliations:** 1 Anesthesia and Intensive Care Medicine, Department of Critical Care, AORN Ospedali Dei Colli, Naples, Italy; 2 Anesthesia and Intensive Care, IRCCS Azienda Ospedaliera Metropolitana, Genoa, Italy; 3 Department of Surgical Sciences and Integrated Diagnostics, University of Genoa, Genoa, Italy; 4 Laboratory of Pulmonary Investigation, Carlos Chagas Filho Institute of Biophysics, Federal University of Rio de Janeiro, Rio de Janeiro, Brazil

**Keywords:** acute respiratory distress syndrome, endotypes, personalized medicine, precision pharmacotherapy, treatable traits

## Abstract

Acute respiratory distress syndrome (ARDS) remains a major cause of morbidity and mortality despite advances in supportive care and decades of unsuccessful pharmacologic investigation. A central limitation has been the treatment of ARDS as a uniform clinical syndrome rather than a biologically heterogeneous condition characterized by distinct mechanisms of injury, repair, and therapeutic responsiveness. Recent advances in biomarker profiling, transcriptomics, and data-driven phenotyping have identified reproducible biological subphenotypes, including hyperinflammatory and hypoinflammatory states associated with differing outcomes and treatment responses. Complementary frameworks such as endotypes and treatable traits offer a more mechanistic approach to stratification by linking specific biological processes, including dysregulated inflammation, endothelial dysfunction, coagulation abnormalities, and impaired epithelial repair, to potential therapeutic targets. In this narrative review, we examine how biological heterogeneity may explain the repeated failure of pharmacologic trials in unselected ARDS populations and critically evaluate emerging targeted therapeutic strategies, including anti-inflammatory, anticoagulant and endothelial-directed, epithelial reparative, and adjunctive or repurposed interventions. We also discuss how treatment response may depend not only on biological phenotype, but on disease stage and temporal evolution. Finally, we examine the clinical and methodological requirements for implementing precision pharmacotherapy in ARDS, including biomarker-guided enrichment, adaptive trial design, and integration of artificial intelligence–driven multimodal data analysis. Although major barriers remain, including biomarker validation, bedside feasibility, and prospective clinical testing, the most credible path forward is unlikely to be a universal therapy for ARDS, but rather biologically and temporally tailored treatment strategies matched to the patients most likely to benefit.

## 1 Introduction

Acute respiratory distress syndrome (ARDS) remains one of the most therapeutically challenging conditions in critical care, with substantial morbidity and mortality despite advances in supportive management (Ashbaugh et al., 1967). Although successive definitions of ARDS, including the American-European Consensus Conference, Berlin, and most recent global definitions, have improved diagnostic consistency and broadened clinical applicability (Bernard et al., 1994; ARDS Definition Task Force et al., 2012; Matthay et al., 2024), they continue to group patients with fundamentally different mechanisms of lung injury under a single clinical diagnosis.

No pharmacological intervention has consistently improved outcomes in unselected patients with ARDS despite decades of experimental research and numerous randomized clinical trials (RCTs) (Meng et al., 2022; Qadir and Chang, 2021; Battaglini et al., 2024). Therapies targeting inflammation, coagulation, endothelial dysfunction, epithelial injury, and other plausible therapeutic pathways have repeatedly failed to demonstrate reproducible clinical benefit. This disconnect suggests that unsuccessful trials may reflect the biological diversity of ARDS as much as the therapies being tested.

Patients meeting identical clinical diagnostic criteria may differ markedly in precipitating etiology, host inflammatory responses, patterns of epithelial and endothelial injury, pulmonary vascular dysfunction, metabolic derangements, and disease trajectory (Sinha and Bos, 2021; Battaglini et al., 2023a; Al-Husinat et al., 2025a). When patients with different disease mechanisms are enrolled within the same trial, treatment effects may be diluted or obscured because the targeted pathway is not uniformly represented across the study population. Consequently, attention has shifted from asking whether a therapy is effective in ARDS to identifying the patients in whom that therapy is most likely to be effective (Battaglini et al., 2022; Al-Husinat et al., 2025b).

These findings support a precision pharmacotherapy approach to ARDS, in which treatment selection is guided by subphenotypes, endotypes, and treatable traits rather than by the clinical syndrome alone.

In this narrative review, we discuss the evidence supporting this approach and examine its implications for drug development, clinical trial design, and implementation in critical care.

## 2 Literature search

This narrative review is based on a focused, non-systematic search of the literature covering experimental, translational, and clinical studies relevant to biological heterogeneity and precision pharmacotherapy in ARDS.

PubMed, MEDLINE, and Scopus were searched for articles published through June 2026 using combinations of Medical Subject Headings (MeSH) and keywords including “acute respiratory distress syndrome,” “ARDS,” “precision medicine,” “precision pharmacotherapy,” “phenotypes,” “subphenotypes,” “endotypes,” “biomarkers,” “hyperinflammatory,” “hypoinflammatory,” “personalized therapy,” “targeted treatment,” and “clinical trials.”

Reference lists of relevant reviews, landmark studies, and international guidelines were screened to identify additional publications. Preference was given to mechanistic and translational studies, randomized controlled trials, high-quality observational studies, consensus statements, and recent reviews that informed current understanding of biological heterogeneity and precision pharmacotherapy in ARDS.

Because this is a narrative review, no formal systematic study selection process or meta-analysis was undertaken.

## 3 Biological heterogeneity in ARDS

A hallmark of ARDS is the fact that presentation of symptoms and biology are dissociated to some degree (Ziaka and Exadaktylos, 2025). Simple physiological and radiographic features are the diagnostic criteria, but they only reflect just part of a very complex and dynamic syndrome (Battaglini et al., 2025). Patients with similar hypoxemia and similar imaging findings may show distinctly different mechanisms for immune activation, tissue injury and repair, highlighting the inadequacy of a purely clinical categorization. A significant step forward in characterizing a biological variability of ARDS has been made through the establishment of reproducible subphenotypes (Battaglini et al., 2026). The differentiation between hyperinflammatory and hypoinflammatory phenotypes has become one of the most valid and clinically significant among these (Calfee et al., 2014; Famous et al., 2017; Sinha et al., 2018; Delucchi et al., 2018; Calfee et al., 2018; Bos et al., 2017).

The hyperinflammatory subphenotype is characterized by elevated circulating inflammatory mediators, greater vasopressor requirements, more severe organ dysfunction, and worse clinical outcomes. In contrast, the hypoinflammatory subphenotype is associated with lower systemic inflammation and a more favorable prognosis. Recent advances in high-dimensional molecular profiling have refined this classification by integrating molecular and physiological characteristics. Biomarker- and transcriptomic-based approaches have improved patient stratification while providing mechanistic insights that support the development of targeted therapeutic strategies. Transcriptomic studies have shown enrichment of innate immune, interferon-related, and neutrophil-driven pathways in the hyperinflammatory subphenotype, whereas hypoinflammatory profiles are more commonly associated with adaptive immune responses and tissue repair pathways (Battaglini et al., 2022; Al-Husinat et al., 2025b; Calfee et al., 2014; Famous et al., 2017; Sinha et al., 2018; Delucchi et al., 2018; Calfee et al., 2018; Bos et al., 2017; Chen et al., 2022; Valda Toro et al., 2024; Sheu et al., 2010; Xu et al., 2025). Incorporating molecular biomarkers into phenotypic classification enhances risk stratification and facilitates mechanism-based therapeutic approaches. Importantly, these subphenotypes also have therapeutic implications. Emerging evidence indicates that biological subgroups differ in their response to standard interventions, including ventilatory strategies, fluid management, and immunomodulatory therapies (Calfee et al., 2014; Famous et al., 2017; Sinha et al., 2018; Delucchi et al., 2018; Calfee et al., 2018; Bos et al., 2017). Patients with hyperinflammatory subphenotype appear more likely to benefit from higher positive end-expiratory pressure (PEEP), restrictive fluid strategies, and corticosteroid therapy, whereas these interventions may provide little benefit—or even be harmful—in other biological contexts. These findings indicate that treatment response depends on the mechanism driving disease rather than on the clinical syndrome alone.

The mechanisms driving ARDS also evolve over time (Al-Husinat et al., 2025a). Inflammatory and exudative functions are observed in the early days, and proliferative and fibrotic reactions are present in the latter stages with great interpopulation diversity in pathophysiology and injury resolution (Ziaka and Exadaktylos, 2025). Temporal alterations in biomarker profiles and gene expression profiles imply that during the illness period patients switch between biological phases, thereby modulating individual response to treatments. This dynamic feature supports the need for continual assessment on a biological level as well as the importance of personalized, as well as durable, therapeutic approaches to therapies throughout life that are time- and space-dependent.

## 4 Endotypes and treatable traits

The identification of biological subphenotypes has substantially improved understanding of heterogeneity in ARDS. However, these classifications remain largely descriptive and do not fully explain the mechanisms that drive disease expression (Battaglini and Schultz, 2026). A critical distinction must therefore be made between subphenotypes, which describe reproducible clinical or biological patterns, and endotypes, which reflect distinct pathobiological mechanisms underlying disease behavior (Merola and Battaglini, 2025). Moving beyond descriptive classification is essential if biologically targeted therapies are to be developed successfully.

The concept of treatable traits offers a more clinically practical approach to translating biological complexity into therapeutic decision-making. Instead of treating ARDS as a uniform syndrome managed with broadly applied interventions, this approach focuses on identifying measurable and potentially modifiable biological abnormalities that may be linked to specific therapies (Bos et al., 2022). This perspective better reflects clinical reality, where patients who fulfill the same diagnostic criteria may nonetheless have fundamentally different dominant mechanisms of injury.

Several biological domains are particularly relevant. Dysregulated inflammation remains one of the most important, ranging from adaptive immune responses to maladaptive hyperinflammatory states associated with organ dysfunction and poor outcomes (Merola et al., 2026). Coagulation abnormalities, including microvascular thrombosis and impaired fibrinolysis, may further contribute to disease progression and represent plausible therapeutic targets (Livingstone et al., 2021). Similarly, the extent of epithelial and endothelial injury reflects disruption of alveolar–capillary barrier integrity and is closely associated with disease severity and progression (Prescott et al., 2016; Bos et al., 2021).

Importantly, these processes rarely occur in isolation. The pathobiology of ARDS is more likely characterized by overlapping and evolving mechanisms than by rigid, mutually exclusive biological categories. Their relative contribution may vary considerably between patients and may also shift during illness, reinforcing the limitations of static classification systems and the need for multidimensional profiling (Bos et al., 2022).

Despite increasing biological resolution, translation into clinical practice remains limited. Reproducibility among patient cohorts is inconsistent, biomarker-based approaches remain insufficiently standardized, and practical bedside tools capable of rapidly identifying relevant biological states are still lacking (Bos et al., 2022). Temporal heterogeneity introduces an additional challenge, as the dominant biological drivers of disease may change over time, potentially altering both mechanistic classification and responsiveness to therapy (Al-Husinat et al., 2025a; Ziaka and Exadaktylos, 2025).

Progress now depends on robust external validation, integration of multimodal biological and clinical data, and the development of pragmatic tools that support mechanism-informed therapeutic decisions in real time. Incorporating treatable traits into dynamic longitudinal models may ultimately provide a more credible biological foundation for precision medicine in ARDS.

## 5 Why pharmacologic trials have failed

Despite extensive investigation and compelling mechanistic rationale, no pharmacologic therapy has consistently improved outcomes in ARDS. Persistent therapeutic failure reflects both the biological complexity of ARDS and longstanding limitations in patient selection, trial design, and treatment timing (Battaglini et al., 2024).

A fundamental limitation of previous ARDS trials has been the assumption that patients meeting the same clinical definition share similar therapeutic targets. Clinical trials based on broad diagnostic criteria inevitably enroll patients with markedly different underlying etiologies, host responses, mechanisms of lung injury, and clinical trajectories (Iwashyna et al., 2015). Therefore, therapies directed at specific biological pathways are unlikely to demonstrate consistent benefit across unselected populations. An intervention that is effective in a biologically defined subgroup may fail to show benefit at the population level when the targeted mechanism is absent or not a dominant driver of disease in a substantial proportion of enrolled patients.

Therapeutic timing has posed a further challenge. Many pharmacologic interventions are intended to modulate biological processes that evolve over the course of illness, yet ARDS has often been approached in clinical trials as though it were a relatively fixed entity. Limited attention to disease phase or temporal shifts in dominant mechanisms increases the likelihood that therapies may be introduced outside the relevant therapeutic window—either after the targeted process is no longer biologically active or before it has become clinically important (Meng et al., 2022; Battaglini et al., 2024).

Conventional randomized trial design has also constrained progress. Traditional studies generally assume relative biological homogeneity and a reasonably consistent treatment response, assumptions that are difficult to reconcile with the marked heterogeneity and dynamic pathobiology of ARDS (Iwashyna et al., 2015). Alternative approaches, including enrichment strategies, biomarker-guided enrollment, and adaptive platform designs, offer a more biologically coherent path forward, but remain insufficiently integrated into ARDS research (Battaglini et al., 2026; Kaizer et al., 2023; Duan et al., 2024; Reddy et al., 2026).

The coronavirus disease 2019 (COVID-19) pandemic offered a revealing counterexample. Although severe COVID-19 frequently fulfilled diagnostic criteria for ARDS, recognition of the underlying disease as a more biologically coherent entity enabled a more targeted therapeutic approach (Bos and Ware, 2022; Gorman et al., 2022). Identification of dysregulated host inflammation as a dominant pathogenic mechanism in selected patients supported the effective use of corticosteroids and immunomodulatory therapies, highlighting how treatment success becomes more likely when therapeutic decisions are guided by defined biological mechanisms rather than syndromic classification alone (Leligdowicz et al., 2022; Meyer et al., 2021; Alhazzani et al., 2021).

Importantly, neutral or negative trials should not be interpreted as evidence that pharmacologic therapy has no role in ARDS. Secondary analyses have repeatedly demonstrated heterogeneity of treatment effect, suggesting that the absence of benefit in the overall study population may conceal clinically meaningful responses in biologically distinct subgroups (Famous et al., 2017; Sinha et al., 2018; Calfee et al., 2018). These findings reinforce the importance of incorporating biological stratification into therapeutic development, as well as into the design, conduct, and interpretation of future trials.

**FIGURE 1:**
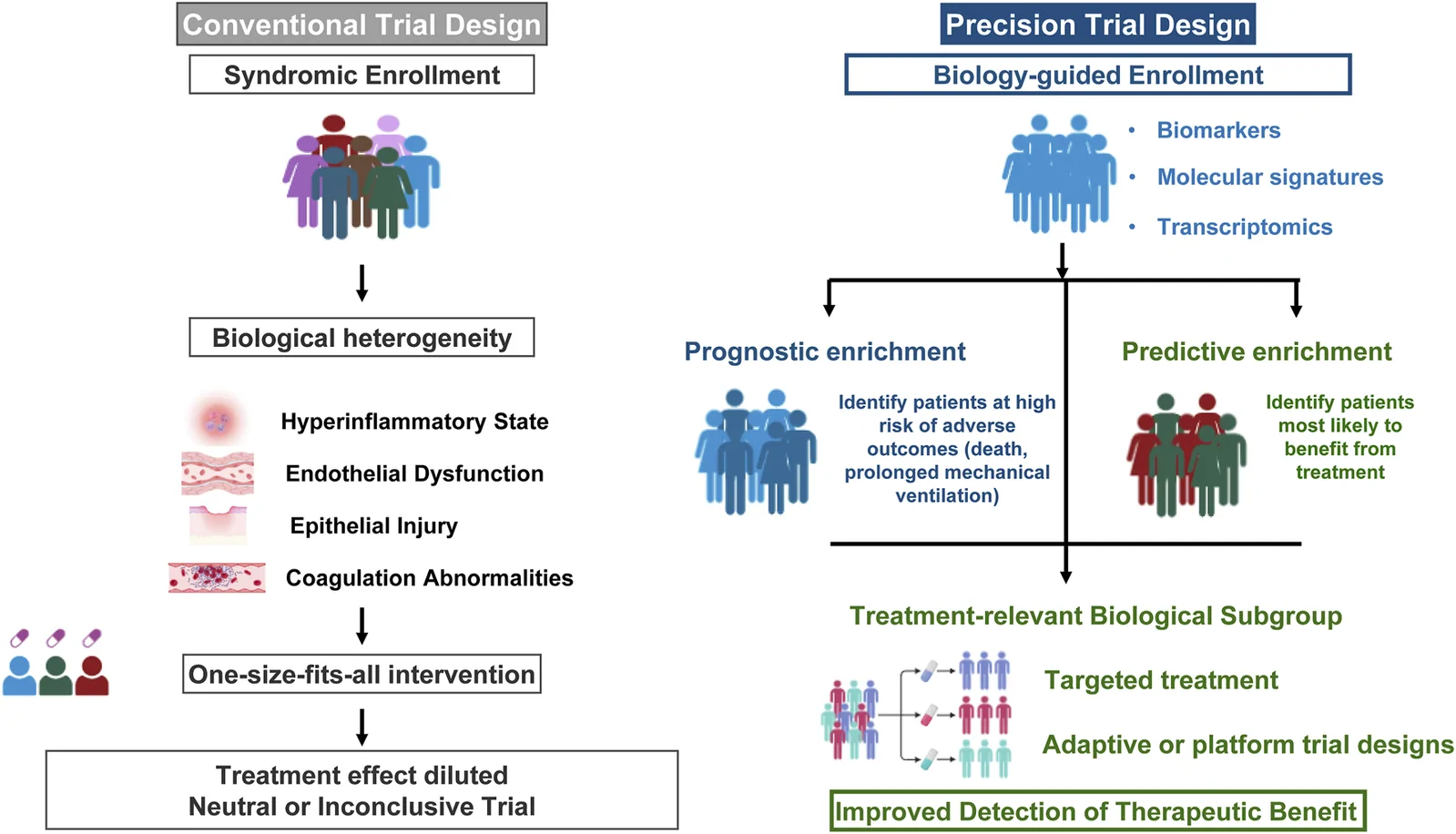
Conventional *versus* precision trial design for pharmacological interventions in acute respiratory distress syndrome (ARDS). Conventional clinical trials typically enroll patients based on syndromic diagnostic criteria, assuming a homogeneous disease population despite substantial underlying biological heterogeneity. This approach may expose biologically distinct patient subgroups to the same intervention, diluting treatment effects and contributing to neutral or inconclusive trial results. In contrast, precision trial designs incorporate biology-guided enrollment using biomarkers, molecular signatures, transcriptomic profiles, or other mechanistic indicators to identify clinically relevant biological subgroups. Prognostic enrichment selects patients at higher risk of adverse outcomes, whereas predictive enrichment identifies those most likely to respond to a specific therapy. These strategies enable targeted pharmacological interventions and facilitate adaptive or platform trial designs, thereby increasing the likelihood of detecting meaningful therapeutic benefit while advancing precision medicine in ARDS.

Taken together, the limited success of pharmacologic trials in ARDS reflects a persistent mismatch between syndromic trial design and the underlying biological heterogeneity of the disease (Figure 1). Future trials should incorporate enrichment strategies and adaptive designs that link patient selection to the mechanism targeted by the intervention.

## 6 Enrichment strategies in ARDS trials

Repeated negative trials in ARDS have exposed a fundamental limitation of conventional study design: broad syndromic enrollment assumes a degree of biological uniformity that does not exist (Ware et al., 2020; Wick et al., 2022). Patients meeting the same clinical definition may have markedly different underlying mechanisms of lung injury, inflammatory responses, and trajectories of disease progression. In this context, failure to detect benefit may reflect biological misalignment between the intervention and the study population rather than lack of therapeutic efficacy.

Enrichment strategies have emerged as one way to address this problem by improving patient selection. Two approaches are commonly distinguished. Prognostic enrichment identifies patients at greater risk of adverse outcomes, such as death or prolonged mechanical ventilation, with the primary aim of increasing event rates and improving trial efficiency. Predictive enrichment addresses a different question: which patients are biologically most likely to respond to the intervention being tested? This strategy is particularly relevant in ARDS, where clinical severity alone offers limited insight into the dominant mechanisms driving disease. By linking enrollment to biological plausibility, predictive enrichment aligns more closely with the principles of precision medicine (Ware et al., 2020; Wick et al., 2022; Kitsios et al., 2019; Reddy et al., 2025).

Advances in molecular profiling have enabled biological stratification beyond conventional clinical classification. Circulating biomarkers, transcriptomic signatures, and related molecular profiles have identified reproducible subgroups characterized by hyperinflammatory states or predominant epithelial and endothelial injury (Battaglini et al., 2022; Al-Husinat et al., 2025b; Calfee et al., 2014; Famous et al., 2017; Sinha et al., 2018; Delucchi et al., 2018; Calfee et al., 2018; Bos et al., 2017; Chen et al., 2022; Valda Toro et al., 2024; Sheu et al., 2010; Xu et al., 2025). These approaches provide a biological basis for selecting patients most likely to respond to targeted interventions. Sinha and colleagues further simplified this strategy by developing parsimonious classifier models that identify hyperinflammatory and hypoinflammatory subphenotypes using a limited number of plasma biomarkers and routinely available clinical variables, eliminating the need for latent class analysis. Validation across independent ARDS cohorts supports their use as practical tools for predictive enrichment in future clinical trials (Sinha et al., 2020). Clinical implementation is further limited by inconsistent reproducibility across cohorts, the lack of standardized biomarker assays, and the difficulty of obtaining biologically informative data within the narrow therapeutic window of critical illness.

Methodological innovation is equally important. Conventional randomized trials are inherently rigid, relying on fixed eligibility criteria and static intervention structures that may be poorly suited to a syndrome characterized by biological variability and temporal evolution. Adaptive trial designs offer greater flexibility, allowing modification of predefined study parameters as data accumulate (Wick et al., 2022; Ben-Eltriki et al., 2024). Platform trials extend this model further by enabling simultaneous evaluation of multiple therapies within a common infrastructure, with interventions added or discontinued as evidence emerges (Vignarajah and Rochwerg, 2024). Such designs are particularly attractive in ARDS, where both disease biology and therapeutic responsiveness may shift over time (Ware et al., 2020; Wick et al., 2022).

Retrospective analyses have provided further evidence that broad syndromic enrollment can obscure clinically relevant signals. In several trials deemed neutral overall, secondary analyses have suggested treatment benefit within biologically or clinically defined subgroups that were not apparent in the primary analysis (Famous et al., 2017; Sinha et al., 2018; Calfee et al., 2018; Spicer et al., 2024). Although these observations remain exploratory, they have been instrumental in generating mechanistic hypotheses and shaping subsequent trial design.

Progress in ARDS therapeutics will likely depend on trial designs that align patient selection with the biological mechanism targeted by the intervention. Enrichment strategies are not a complete solution, but they represent an essential move toward more rational therapeutic evaluation.

## 7 Targeted pharmacologic strategies

### 7.1 Anti-inflammatory therapies

Inflammation has long been considered a central therapeutic target in ARDS, although the clinical experience with immunomodulatory therapy has been far less straightforward than the biological rationale would suggest (Merola et al., 2026; Wick et al., 2022). Compelling mechanistic rationale has repeatedly failed to translate into consistent clinical benefit, underscoring the complexity of inflammatory signaling in a biologically heterogeneous syndrome. Corticosteroids remain the most extensively studied anti-inflammatory agents in ARDS, with outcomes shaped heavily by timing, disease stage, and patient selection (Chaudhuri et al., 2024; Qadir et al., 2024; Soumare et al., 2026; Guglielmi et al., 2026).

Their biological effects are well established, including suppression of pro-inflammatory cytokine signaling, attenuation of neutrophil activation, reduction of oxidative stress, and limitation of inflammatory tissue injury (Hong et al., 2022; Rhen and Cidlowski, 2005; Landolf et al., 2022; Annane et al., 2017).

Clinical results, however, have been inconsistent. Earlier trials frequently failed to demonstrate benefit and, in some cases, raised concern regarding potential harm, particularly when treatment was initiated late or in patients with more established fibroproliferative lung injury (Verchère et al., 2025; Meduri et al., 1998; Annane et al., 2006).

Part of this inconsistency likely reflects the temporal biology of ARDS. The inflammatory processes that dominate early in the syndrome are not necessarily those that drive later phases of lung injury, yet many earlier corticosteroid studies treated ARDS as a relatively static entity. This may explain some of the conflicting results. More recent investigations have placed greater emphasis on earlier intervention, with more consistent signals of benefit.

The DEXA-ARDS trial by Villar and colleagues represented an important turning point. In patients with moderate-to-severe ARDS, early dexamethasone administration increased ventilator-free days and reduced short-term mortality. These findings differed from several earlier neutral studies and reinforced the view that timing is a major determinant of corticosteroid efficacy (Villar et al., 2020).

More recently, a dose-response network meta-analysis reported that dexamethasone and methylprednisolone were associated with the greatest survival benefit when administered at near-maximal effective doses, whereas pulse-dose regimens did not provide additional advantages. The study also confirmed improvements in ventilator-free days, supporting the use of appropriately dosed corticosteroid therapy in both COVID-19 and non-COVID-19 ARDS, although the overall certainty of evidence remained low to moderate (Imai et al., 2025).

Current Society of Critical Care Medicine guidelines support the use of corticosteroid in selected critically ill patients with ARDS, while acknowledging persistent uncertainty regarding the optimal agent, dose, duration, timing, and the patient populations most likely to benefit (Chaudhuri et al., 2024). The evidence does not support corticosteroids as a universal intervention for ARDS; rather, any benefit appears to depend on alignment between treatment, biological phenotype, and disease stage.

Interest has also shifted toward more selective immunomodulatory strategies. The appeal is clear: if broad immunosuppression produces variable results, targeting specific inflammatory pathways may offer greater biological precision.

Interleukin-1 blockade with anakinra has shown encouraging signals in selected populations, particularly in COVID-19-associated respiratory failure, where hyperinflammatory phenotypes may be more readily identifiable (Cavalli et al., 2020).

Interleukin-6 receptor inhibition with tocilizumab has likewise been associated with improved outcomes in critically ill COVID-19 patients, particularly when combined with corticosteroids (Soin et al., 2021; Recovery Collaborative Group, 2021; REMAP-CAP Investigators, 2021; WHO Rapid Evidence Appraisal for COVID-19 Therapies REACT Working Group, 2021).

The COVID-19 pandemic provided an important proof of principle for precision pharmacotherapy. Although severe COVID-19 frequently fulfilled the clinical criteria for ARDS, it was characterized by a more homogeneous and better-defined inflammatory pathobiology than syndromically classified ARDS. The clinical benefit observed with immunomodulatory therapies likely reflected not only the efficacy of the interventions themselves but also a closer alignment between therapeutic strategy, disease stage, and the predominant biological mechanisms. These observations have important implications for ARDS, supporting the concept that successful pharmacological treatment depends on matching therapies to the underlying biology rather than to the clinical syndrome alone.

Not all mechanistically plausible anti-inflammatory strategies have translated into clinical benefit. Tumor necrosis factor alpha (TNF-α) inhibition, despite compelling experimental rationale, has failed to demonstrate consistent efficacy in sepsis-associated critical illness (Bertok et al., 2012; Abraham et al., 1995; Abraham et al., 1998). This remains a cornerstone of ARDS treatment: mechanistic plausibility alone is not enough.

Inflammation should not be considered a uniform therapeutic target in ARDS. The critical question is no longer whether inflammation should be treated, but which inflammatory pathway should be targeted, in whom, and at what stage of disease (Huang et al., 2024; Pensier et al., 2025).

### 7.2 Anticoagulant and endothelial-targeted therapies

Coagulation abnormalities and endothelial injury are now recognized as fundamental components of ARDS pathobiology rather than merely downstream consequences of inflammation (Vassiliou et al., 2020). Activation of coagulation pathways, impaired fibrinolysis, and pulmonary microvascular thrombosis are hallmark features of the syndrome that contribute to alveolar–capillary barrier disruption, increased vascular permeability, ventilation–perfusion mismatch, and progressive hypoxemia (Livingstone et al., 2021; Vassiliou et al., 2020). However, the contribution of these mechanisms likely varies across biological subgroups. In patients in whom thrombo-inflammatory and endothelial dysfunction predominate, these pathways may represent biologically relevant therapeutic targets (Bos et al., 2022). This rationale has supported sustained interest in anticoagulant and endothelial-directed therapies, although clinical results have been disappointing.

Heparin has been one of the most extensively explored agents, in part because its biological effects extend beyond anticoagulation. Experimental data suggest anti-inflammatory properties, including modulation of inflammatory signaling, attenuation of neutrophil-mediated injury, and reduced fibrin deposition within the alveolar space (Xiao et al., 2024). Both systemic and inhaled formulations have therefore been investigated. Early studies of nebulized heparin reported encouraging physiological signals, including improved oxygenation and attenuation of lung injury progression, with an acceptable safety profile (Dixon et al., 2010; van Haren et al., 2025). Some reports also suggested reduced progression to established ARDS in mechanically ventilated patients at risk (Dixon et al., 2021). These findings, however, have not translated into consistent improvements in survival or other clinically meaningful outcomes. As with many therapeutic strategies in ARDS, interpretation is complicated by heterogeneity in disease severity, timing of intervention, and uncertainty regarding the extent to which thrombo-inflammatory pathways drive disease in individual patients (Suzuki et al., 2020).

Strategies aimed at endothelial protection have followed a similar course. Recombinant human thrombomodulin emerged as a biologically attractive candidate because of its anticoagulant and endothelial-protective properties. Preclinical studies demonstrated attenuation of inflammatory injury, preservation of endothelial integrity, and improved survival in experimental ARDS models (Fresilli et al., 2026; Hirata et al., 2021). Yet these mechanistic signals were not reproduced in randomized clinical trials (Vincent et al., 2019).

Other vascular-directed approaches have yielded similarly inconsistent results. Recombinant angiotensin-converting enzyme 2, prostacyclin-based therapies, and nitric oxide-related interventions have shown physiological effects in selected settings, but convincing improvements in patient-centered outcomes remain lacking (Khan et al., 2017; Torbic et al., 2023; Redaelli et al., 2023; Brojakowska et al., 2020). This recurring pattern reflects a broader challenge in ARDS therapeutics: biologically plausible interventions often fail when evaluated in clinically defined populations in whom the targeted pathway is neither uniformly present nor biologically dominant.

The limited success of these strategies should not be interpreted as evidence that vascular biology is therapeutically unimportant in ARDS (Fresilli et al., 2026). A more likely explanation is inadequate biological stratification. Therapies directed at coagulation or endothelial dysfunction are unlikely to provide uniform benefit across a heterogeneous syndrome. Their role may be more relevant in patients with marked endothelial activation, hypercoagulability, or microvascular injury as dominant mechanisms of disease. Progress in this area will likely depend on more precise phenotyping to identify patients in whom endothelial dysfunction or thrombo-inflammatory injury represents a dominant mechanism rather than a secondary consequence of critical illness.

### 7.3 Epithelial and alveolar repair strategies

Loss of alveolar epithelial integrity is a defining feature of ARDS and a major contributor to persistent respiratory failure and delayed recovery (Esquivel-Ruiz et al., 2021). Injury to both type I and type II alveolar epithelial cells disrupts the alveolar–capillary barrier, impairs surfactant homeostasis and alveolar fluid clearance, and perpetuates pulmonary edema, worsening gas exchange while limiting endogenous repair (Wang and Chao, 2025). These mechanisms place epithelial dysfunction at the center of ARDS pathobiology and provide a strong biological rationale for therapies aimed at restoring epithelial integrity and promoting lung repair.

Mesenchymal stromal cell (MSC)-based therapies initially generated considerable enthusiasm because of their combined immunomodulatory and reparative properties (Khoury et al., 2020; Abreu et al., 2021; Wu et al., 2025). Early randomized studies established an acceptable safety profile across ARDS populations (Matthay et al., 2019; Bellingan et al., 2022). Clinical efficacy, however, has remained elusive. A subsequent phase 2b randomized trial failed to demonstrate meaningful benefit in patients with moderate-to-severe ARDS, including COVID-19-associated respiratory failure (Matthay et al., 2026). These findings tempered early optimism and exposed practical limitations inherent to live-cell therapy, including inconsistent engraftment, limited persistence, and manufacturing complexity (da Silva et al., 2025).

Interest has increasingly shifted toward mesenchymal stromal cell-derived extracellular vesicles (MSC-EV) as a cell-free alternative. By carrying bioactive mediators implicated in immunoregulation and tissue repair, these vesicles may preserve key biological effects of parent cells while offering greater therapeutic standardization (Costa-Ferro et al., 2024; Couto et al., 2025). Early clinical studies suggest acceptable tolerability and biological activity following intravenous or inhaled administration, with preliminary signals of anti-inflammatory effects and possible clinical benefit (Zarrabi et al., 2023; Zhang et al., 2022). Whether these findings will translate into reproducible clinical efficacy remains uncertain. Standardization of vesicle production, characterization, potency assessment, and dosing remain a substantial barrier.

Growth factor–based strategies have been explored to promote alveolar epithelial repair. Granulocyte–macrophage colony-stimulating factor (GM-CSF) has key roles in surfactant homeostasis, innate immunity, and epithelial regeneration (Qadir and Chang, 2021). Experimental data suggest protective effects against epithelial injury, and observational studies have associated higher bronchoalveolar concentrations with improved outcomes in ARDS (Paine et al., 2003; Matute-Bello et al., 2000). Clinical evidence has been less consistent. Intravenous GM-CSF failed to improve major clinical outcomes in randomized trials, although modest reductions in organ support requirements have been reported (Paine et al., 2012; Sun et al., 2023). By contrast, small studies of inhaled GM-CSF have shown improvements in oxygenation, potentially through restoration of alveolar macrophage function, enhanced epithelial repair, and improved surfactant homeostasis. Whether these physiological effects translate into meaningful clinical benefit remains uncertain, as available studies are small and have not demonstrated consistent improvements in mortality or other patient-centered outcomes (Herold et al., 2014). Larger randomized trials will be needed to determine whether inhaled GM-CSF benefits biologically selected patients with ARDS.

Restoration of alveolar fluid clearance represents another biologically plausible strategy. Under physiological conditions, active epithelial sodium and chloride transport drives fluid reabsorption from the alveolar space. In ARDS, epithelial injury and inflammatory disruption frequently impair this mechanism, contributing to persistent pulmonary edema (Taenaka and Matthay, 2025). β-adrenergic agonists were initially considered promising because of their capacity to enhance epithelial sodium transport and accelerate fluid clearance, findings consistently supported in experimental models and *ex vivo* human lungs (Taenaka and Matthay, 2025; Pittet et al., 1994; Vivona et al., 2001; Frank et al., 2003; Wolk et al., 2008). Clinical translation, however, was disappointing. Large, randomized trials failed to demonstrate benefit and, in some cases, identified excess adverse events (Gates et al., 2013; Festic et al., 2017). More recently, solnatide, an inhaled peptide that directly activates epithelial sodium channels, has emerged as a more targeted approach. Early preclinical and phase 1–2 studies are encouraging, although its therapeutic role remains undefined (Schmid et al., 2021).

A conceptually distinct strategy involves restoration of immune competence in patients with persistent ARDS characterized by immune exhaustion. Prolonged critical illness may be accompanied not only by unresolved epithelial injury but also by profound adaptive immune dysfunction, including T-cell exhaustion mediated through checkpoint pathways such as programmed cell death protein 1 and programmed death ligand 1 (Leligdowicz et al., 2022). Experimental studies suggest that checkpoint inhibition may restore host immune competence, improve pathogen clearance, and potentially influence resolution of lung injury in selected biological contexts (Leligdowicz et al., 2022). Clinical experience in ARDS remains extremely limited, and the risk of exacerbating injurious inflammation is an important concern. Nonetheless, this line of investigation reflects a broader shift in therapeutic thinking: in selected patients, restoration of immune competence may be as relevant as suppression of inflammation.

The effectiveness of epithelial reparative therapies may depend on timing. Interventions designed to limit early epithelial injury differ fundamentally from those intended to promote later repair or restore immune competence. The greatest opportunity for clinical benefit lies in patients in whom epithelial dysfunction remains a dominant and potentially reversible mechanism of disease.

### 7.4 Adjunctive and repurposed therapies

A wide range of adjunctive and repurposed pharmacological therapies has been investigated in ARDS based on plausible anti-inflammatory, immunomodulatory, antioxidant, or endothelial-protective mechanisms (Qadir and Chang, 2021; Battaglini et al., 2024). Despite this strong biological rationale, most have failed to demonstrate consistent clinical benefit. This recurring pattern likely reflects not only the limitations of individual interventions but also the evaluation of mechanistically targeted therapies in clinically defined populations with substantial biological heterogeneity. Consequently, treatment effects are likely to be diluted when the targeted pathway is not a dominant driver of disease across the enrolled population.

Statins remain among the most extensively studied examples. Their biological effects extend well beyond lipid lowering, with recognized actions on endothelial function, inflammatory signaling, and coagulation pathways (Battaglini et al., 2024). Early mechanistic studies generated enthusiasm. In the HARP trial, simvastatin was associated with reductions in systemic inflammation and organ dysfunction. Those signals did not translate into improved clinical outcomes in the larger HARP-2 trial, which found no benefit in ventilator-free days, extrapulmonary organ failure-free days, or mortality (Craig et al., 2011; McAuley et al., 2014).

Subsequent analyses suggested that the neutral overall findings did not fully capture biologically distinct treatment responses. In a *post hoc* analysis of the HARP-2 trial, simvastatin was associated with improved survival among patients with the hyperinflammatory ARDS subphenotype (Calfee et al., 2018). By contrast, rosuvastatin failed to improve clinical outcomes in the SAILS trial, and secondary analyses provided only limited evidence of phenotype-dependent benefit (Sinha et al., 2018; National Heart et al., 2014). Whether these divergent findings reflect pharmacological differences between statins, differences in the biological characteristics of the enrolled populations, or limitations of current phenotyping strategies remains uncertain (Gorman et al., 2022).

Biomarker-based analyses have added another layer of complexity. Elevated interleukin-18 has emerged as a marker of more severe inflammatory injury and worse prognosis (Moore et al., 2023). Importantly, high concentrations have also been identified in patients outside conventionally defined hyperinflammatory phenotypes, suggesting that latent class models may not fully capture therapeutically relevant biology. Simvastatin appeared more effective in patients with higher baseline interleukin-18 levels, raising the possibility that biomarker-guided selection may offer greater precision than broader phenotypic classification alone (Boyle et al., 2022).

Other repurposed therapies have followed a similar trajectory. Vitamin C, vitamin D, N-acetylcysteine, interferons, aspirin, and related antioxidant or immunomodulatory agents have repeatedly shown biological plausibility without convincing clinical efficacy (Battaglini et al., 2024). Interferon-β improved physiological parameters in early studies but failed to reduce ventilator dependence or mortality in later randomized trials (Meyer et al., 2021; Bellingan et al., 2014; Ranieri et al., 2020). Trials of vitamin C and vitamin D have likewise been largely neutral despite compelling mechanistic rationale related to oxidative stress, endothelial integrity, and immune regulation (National Heart et al., 2019; Fowler et al., 2019; Zhang et al., 2017; Fowler, 2024). N-acetylcysteine has not demonstrated survival benefit, and aspirin has failed to improve either prevention or treatment outcomes (Kor et al., 2016; Toner et al., 2022; Bernard et al., 1997).

Neuromuscular blocking agents occupy a somewhat different position. Although traditionally regarded as supportive therapy, their effects may extend beyond facilitation of lung-protective ventilation. Experimental and translational studies suggest potential immunomodulatory effects, including attenuation of inflammatory signaling, particularly with cisatracurium (Qadir et al., 2024). Current Society of Critical Care Medicine guidelines suggest their use in adults with ARDS and persistent hypoxemia (PaO_2_/FiO_2_ <150) or failure to achieve lung-protective ventilation targets despite optimized sedation and ventilator management, with use generally limited to short courses not exceeding 48 h (Erstad et al., 2026). Emerging evidence also suggests that response may vary by physiological phenotype. A recent analysis found a substantially higher probability of benefit in patients with elevated respiratory system elastance, raising the possibility that patients with more severe mechanical impairment may represent a biologically relevant responder subgroup (Zalucky et al., 2025). Whether any benefit reflects improved mechanics, attenuation of patient self-inflicted lung injury, immunomodulation, or some combination of these mechanisms remains uncertain.

Negative trials should not be interpreted as evidence that the targeted pathways are unimportant, but rather as a reminder of the limitations of syndromic trial design in a complex syndrome. Some of these therapies may yet prove beneficial, but likely in more narrowly defined patient populations selected according to relevant pathophysiological features rather than clinical syndrome alone (Battaglini et al., 2023b). These therapies target distinct components of ARDS pathophysiology, reinforcing the need to align treatment with the dominant drivers of disease in individual patients. A summary of the principal precision pharmacotherapeutic strategies, their biological rationale, current clinical evidence, and key implementation challenges is provided in Table 1.

**TABLE 1:** Biological rationale, clinical evidence, and precision medicine considerations for pharmacological therapies in acute respiratory distress syndrome.

Therapeutic strategy	Representative therapies	Biological rationale	Clinical evidence	Precision medicine considerations
Anti-inflammatory therapies	Corticosteroids (dexamethasone, methylprednisolone)	Suppression of dysregulated inflammation, reduction of cytokine production, attenuation of neutrophil-mediated lung injury	Improved ventilator-free days and short-term survival when initiated early; earlier trials yielded inconsistent results	Benefit depends on disease stage, inflammatory subphenotype, and early treatment
	IL-1 receptor antagonism (anakinra)	Targeted modulation of hyperinflammatory host response	Benefit observed primarily in hyperinflammatory COVID-19 populations	Non-COVID applicability remains uncertain
	IL-6 receptor blockade (tocilizumab)	IL-6 signaling blockade	Improved outcomes in critically ill COVID-19 patients, particularly when combined with corticosteroids; extrapolation to non-COVID ARDS remains uncertain	Greatest potential in hyperinflammatory subphenotypes; infection risk and applicability beyond COVID-19 remain uncertain
Coagulation and endothelial-targeted therapies	Heparin (systemic, inhaled)	Modulation of coagulation, reduction of thrombo-inflammatory injury, and endothelial protection	Improved oxygenation in early studies; consistent survival benefit has not been demonstrated	Therapeutic relevance may be greatest in patients with marked hypercoagulability or endothelial dysfunction
	Recombinant human thrombomodulin	Anticoagulant, anti-inflammatory, endothelial stabilization	Strong mechanistic rationale; randomized trials failed to demonstrate consistent clinical benefit	May benefit patients with predominant endothelial injury
Epithelial repair and regenerative therapies	ACE2-, prostacyclin-, and nitric oxide-targeted therapies	Endothelial protection, vascular tone modulation, microcirculatory support	Physiological benefits observed in selected settings; no consistent improvement in patient-centered outcomes	Limited by imprecise patient selection and vascular heterogeneity
	MSC	Immunomodulation, promotion of epithelial repair	Early phase trials demonstrated safety but not consistent efficacy	Translational barriers include manufacturing complexity, dosing uncertainty, and limited persistence
	MSC-EV	Cell-free immunomodulatory and reparative signaling	Early translational and clinical signals are encouraging; definitive efficacy data are lacking	Standardization, potency assessment, and scalable production remain unresolved
	GM-CSF	Promotion of epithelial repair, surfactant homeostasis, innate immune support	Strong mechanistic rationale; clinical evidence remains limited	Response appears to depend on timing and disease biology
	β-agonists/solnatide	Enhancement of alveolar fluid clearance through epithelial sodium transport	β-agonists failed despite strong mechanistic rationale; solnatide remains investigational	Potentially most effective in early disease with preserved epithelial function
Adjunctive and repurposed pharmacologic therapies	Statins (simvastatin, rosuvastatin)	Endothelial modulation, immunomodulatory effects, anti-inflammatory signaling	No overall benefit in broad ARDS populations; secondary analyses suggest possible benefit in hyperinflammatory or biomarker-defined subgroups	Highlights the importance of biological enrichment and biomarker-guided therapy

Summary of major pharmacologic and adjunctive therapies investigated in acute respiratory distress syndrome (ARDS), organized according to biological rationale, current clinical evidence, and precision medicine considerations. Therapeutic responses are unlikely to be uniform across clinically defined ARDS populations and may depend on biological phenotype, disease stage, and timing of intervention. Evidence derived from coronavirus disease 2019 (COVID-19)-associated ARDS is indicated where relevant; extrapolation to non-COVID ARDS should be interpreted cautiously. Abbreviations: ACE2, angiotensin-converting enzyme 2; ARDS, acute respiratory distress syndrome; COVID-19, coronavirus disease 2019; DEXA-ARDS, Dexamethasone Treatment for the Acute Respiratory Distress Syndrome trial; GM-CSF, granulocyte–macrophage colony-stimulating factor; IL, interleukin; MSC, mesenchymal stromal cell; MSC-EV, mesenchymal stromal cell-derived extracellular vesicle; RCT, randomized controlled trial.

## 8 Timing matters: the importance of disease stage

A persistent challenge in ARDS therapeutics is the assumption that the syndrome represents a biologically static condition. In reality, the mechanisms driving lung injury evolve throughout the course of illness, and those that predominate during early respiratory failure often differ from those sustaining persistent disease or promoting recovery (Sinha and Bos, 2021; Al-Husinat et al., 2025a; Ziaka and Exadaktylos, 2025; Xu et al., 2023). This dynamic biology has important therapeutic implications: an intervention may be mechanistically sound yet fail if the pathway it targets is no longer a dominant driver of disease when treatment is initiated.

The early phase of ARDS is typically characterized by exudative lung injury and pronounced innate immune activation. Cytokine release, neutrophil recruitment, disruption of endothelial and epithelial barriers, alveolar edema, and activation of thrombo-inflammatory pathways predominate during this phase (Ziaka and Exadaktylos, 2025; Merola et al., 2026; Xu et al., 2023). These mechanisms provide the biological rationale for anti-inflammatory and endothelial-directed therapies. Their therapeutic relevance, however, may be transient. Once substantial tissue injury is established, interventions aimed at early inflammatory pathways may have limited capacity to alter the clinical course.

As respiratory failure persists, the dominant mechanisms often shift. Ongoing epithelial injury, fibroblast activation, extracellular matrix deposition, and defective alveolar repair become increasingly important, particularly in patients requiring prolonged ventilatory support (Ziaka and Exadaktylos, 2025; Xu et al., 2023). At this stage, suppressing inflammation may become less relevant and, in some cases, interfere with reparative processes. Interventions aimed at promoting epithelial recovery or restoring alveolar function may be more appropriate. Importantly, these transitions do not follow a predictable timeline. Overlap between inflammatory and reparative pathways is common, and the pace of progression varies substantially between patients.

**FIGURE 2:**
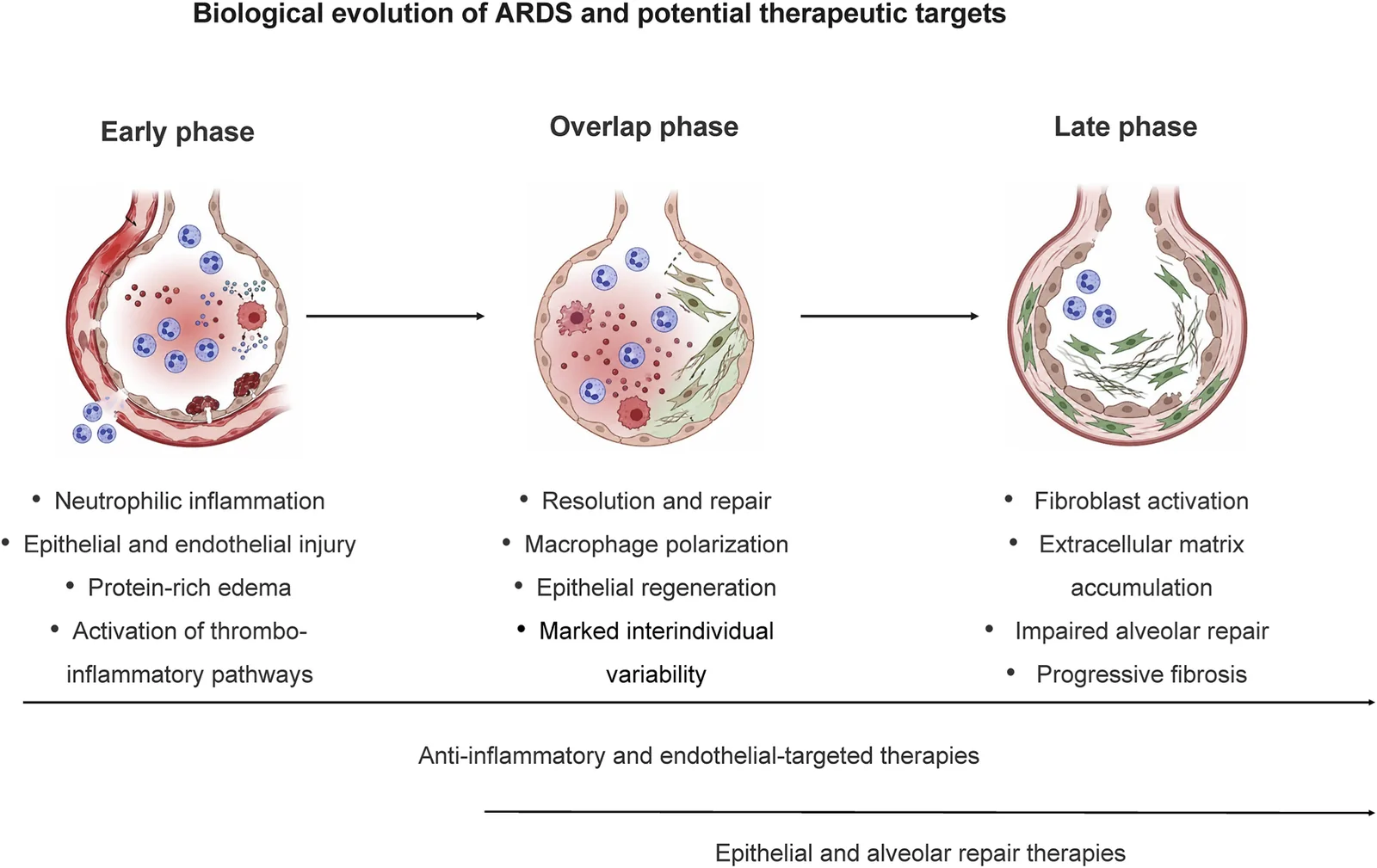
Biological evolution of acute respiratory distress syndrome (ARDS) and stage-specific therapeutic opportunities. ARDS is characterized by dynamic and overlapping biological phases in which the dominant mechanisms of lung injury and repair evolve over time, creating distinct but non-exclusive therapeutic opportunities. The early phase is dominated by exudative lung injury, neutrophilic inflammation, endothelial and epithelial barrier disruption, and protein-rich alveolar edema, providing the principal biological rationale for anti-inflammatory and endothelial-directed therapies. As the disease progresses, an overlap phase emerges in which inflammatory resolution and tissue repair coexist, with macrophage polarization, epithelial regeneration, and substantial interindividual variability in disease progression. During the late phase, persistent fibroblast activation, extracellular matrix accumulation, and progressive fibrosis become increasingly important, supporting therapeutic strategies aimed at restoring epithelial integrity and promoting alveolar repair. The proposed therapeutic windows are conceptual and emphasize that treatment selection should be guided by the predominant biological mechanisms and their temporal evolution rather than by chronological time or a uniform syndrome-based approach.

Chronological time from symptom onset or ARDS diagnosis is therefore an imprecise guide to treatment selection. Two patients enrolled within the same protocol-defined treatment window may be in markedly different mechanistic states. Host-response patterns add further complexity. A patient with early hyperinflammatory ARDS may plausibly benefit from corticosteroids or cytokine-directed therapy, whereas another with limited inflammatory activation or evolving fibroproliferative injury may derive little benefit from the same intervention while remaining exposed to treatment-related harm (Figure 2) (Meduri, 1996).

This temporal dimension contributes to the repeated failure of pharmacologic trials in ARDS. Enrollment has often been determined by fixed chronological criteria rather than by the underlying pathophysiology most relevant to treatment response. A precision approach will require a more dynamic therapeutic framework informed not only by baseline phenotyping, but also by serial assessment of evolving host-response patterns. Longitudinal evaluation of inflammatory, endothelial, epithelial, and fibroproliferative markers may prove more informative than reliance on static syndromic classification alone (Pensier et al., 2025).

## 9 Bedside implementation of precision pharmacotherapy

Although biological heterogeneity in ARDS is increasingly recognized, translating that knowledge into bedside therapeutic decisions remains difficult (Bos et al., 2022). The principal obstacle is practical rather than conceptual. In the intensive care unit, treatment decisions often need to be made within hours, whereas most current approaches to biological stratification rely on biomarker panels, transcriptomic profiling, or analytical methods that are not available in real time (Battaglini et al., 2022; Al-Husinat et al., 2025b; Bos et al., 2022). A precision strategy has little clinical value if the relevant information cannot be obtained quickly enough to influence management.

Robust biomarkers are essential for the clinical implementation of precision pharmacotherapy. Among the most consistently associated with hyperinflammatory subphenotypes and adverse outcomes are interleukin-6, soluble tumor necrosis factor receptor 1, interleukin-18, and C-C motif chemokine ligand 7 (Calfee et al., 2014; Famous et al., 2017; Sinha et al., 2018; Delucchi et al., 2018; Calfee et al., 2018; Bos et al., 2017). Angiopoietin-2 has been linked to endothelial injury and vascular permeability, whereas soluble receptor for advanced glycation end-products, surfactant protein D, and Krebs von den Lungen-6 have been associated with alveolar epithelial damage (Qiao et al., 2024; Hendrickson and Matthay, 2018; Atmowih et al., 2023). Most of these biomarkers have demonstrated prognostic value, whereas evidence supporting their use for therapeutic selection remains limited. Establishing that a biomarker identifies patients who benefit from a specific therapy requires substantially stronger evidence than demonstrating prognostic value alone. Whether they can reliably guide treatment decisions in routine clinical practice remains uncertain. A more practical near-term strategy may be to integrate selected biomarkers with routinely available clinical and physiological variables rather than rely exclusively on molecular profiling.

Precision management in ARDS cannot be reduced to pharmacologic therapy alone. Biological heterogeneity appears to influence responses not only to drug interventions, but also to ventilation strategies, fluid management, and other core components of supportive care (Calfee et al., 2014; Famous et al., 2017; Sinha et al., 2018; Delucchi et al., 2018; Calfee et al., 2018; Bos et al., 2017). Patients with hyperinflammatory profiles, for example, may respond differently to higher PEEP, conservative fluid strategies, or corticosteroids than patients in whom inflammation is not a dominant feature (Merola et al., 2025a; Merola et al., 2025b). Precision care in ARDS should therefore extend beyond drug selection to the broader therapeutic strategy.

Several barriers continue to limit clinical implementation. Biological classifications are not standardized, validated thresholds for treatment allocation remain unavailable, and reproducibility across cohorts is variable (Bos et al., 2022). Even when informative biomarkers exist, practical limitations are considerable, including assay turnaround time, cost, workflow integration, and the infrastructure required to support interpretation (Rizzo et al., 2023). An additional complexity is that the biological profile of ARDS is not static. Host responses evolve over time, so a single biological assessment may no longer reflect the mechanisms driving disease at the time treatment decisions are made (Ma et al., 2025).

For precision pharmacotherapy to become clinically actionable in ARDS, biological stratification must fit the realities of intensive care practice: rapid, practical, and reproducible. Identifying patient subgroups is only the first step; clinicians also need tools that provide this information early enough to guide therapeutic decisions.

## 10 The role of artificial intelligence and data-driven approaches

The growing availability of clinical, physiological, and molecular data has exposed the limitations of conventional statistical methods for studying ARDS, where multiple biological processes interact simultaneously (Lombardi et al., 2026). Artificial intelligence and machine learning allow these complementary sources of information to be analysed together, providing a basis for more refined patient stratification.

Machine learning has been instrumental in defining reproducible ARDS subphenotypes. Unsupervised methods, including latent class analysis and clustering techniques, have consistently identified patient groups with distinct inflammatory profiles, clinical characteristics, and outcomes (Calfee et al., 2014; Famous et al., 2017; Sinha et al., 2018; Delucchi et al., 2018; Calfee et al., 2018; Bos et al., 2017). More recent studies have combined biomarkers, physiological variables, clinical features, and transcriptomic data, allowing a broader description of disease biology than any individual dataset alone (Battaglini et al., 2022; Al-Husinat et al., 2025b; Lombardi et al., 2026). This information may help enrich future pharmacotherapy trials with patients most likely to respond to targeted interventions.

The potential applications of machine learning extend beyond subphenotypic classification. Predictive models estimating treatment response, disease trajectory, or clinical deterioration may also assist therapeutic decision-making, particularly in the time-sensitive setting of critical care (Li et al., 2025). Because these models can incorporate longitudinal information, predictions may evolve as the patient’s condition changes rather than relying solely on baseline assessment.

Translation into routine practice remains limited. Most published models have been derived retrospectively from highly selected cohorts, and only a minority has undergone external validation. Additional limitations include interpretability, data quality, algorithmic bias, and integration into routine ICU workflows (Lombardi et al., 2026). Robust performance across different patient populations and healthcare settings has yet to be demonstrated.

Artificial intelligence is unlikely to replace clinical judgment. Its contribution lies in integrating complex datasets into information that can support therapeutic decisions and improve patient selection. Whether this translates into better outcomes will depend on prospective validation and successful implementation in routine critical care practice.

## 11 Future directions and research agenda

Advancing ARDS pharmacotherapy requires moving beyond trial designs based solely on syndromic definitions toward strategies that reflect the underlying mechanisms of disease. Broad enrollment based solely on clinical diagnostic criteria has repeatedly limited the ability to detect meaningful treatment effects, particularly when interventions target mechanisms that are relevant only in selected patient subsets. Future trials will need to incorporate prospective enrichment strategies, including biomarker-based selection, molecular phenotyping, and characterization of dynamic host-response states. Adaptive and platform trial designs may be particularly well suited to this setting, allowing simultaneous evaluation of multiple interventions while accommodating biological heterogeneity and temporal evolution.

At the same time, a more precise therapeutic framework will require stronger mechanistic foundations. Although subphenotypes and treatable traits have improved molecular characterization of ARDS, much of the current literature remains associative rather than mechanistically definitive. Identifying reproducible patient groups is an important step, but classification alone is not sufficient to support mechanism-based therapy. A more fundamental challenge is establishing clearer links between specific pathobiological pathways and differential therapeutic response. Translating mechanistic insights into effective therapies requires closer integration of translational science and clinical investigation, together with longitudinal biological sampling, experimental models, and multimodal analytical approaches that link molecular signatures to treatment responsiveness.

The implementation of precision medicine in ARDS will also raise practical, regulatory, and ethical challenges. Biomarkers intended to guide treatment allocation will require rigorous validation, reproducibility across diverse populations, and clinically feasible turnaround times. Access to advanced diagnostic tools remains uneven, raising concerns that precision strategies could widen existing disparities in critical care delivery if implementation is restricted to highly resourced settings. Regulatory frameworks will also need to adapt to therapies directed at biologically defined subgroups rather than conventionally defined syndromic populations.

The challenge is no longer generating biological insight, but translating it into decisions that improve patient care. Precision medicine will succeed only if it can be implemented rapidly, practically, and reliably at the bedside.

## 12 Conclusion

Precision pharmacotherapy has shifted the focus of ARDS research from discovering therapeutic targets to selecting the patients most likely to benefit from them. This transition has been driven by advances in molecular phenotyping, together with enrichment strategies, biomarker-guided enrollment, adaptive trial designs, and data-driven analytical approaches. The remaining challenge is implementation. Classification systems require broader validation, biomarkers must become rapid and accessible, and precision strategies need to fit the realities of critical care. Improving outcomes in ARDS requires matching existing and emerging therapies to the right patient at the right stage of disease.
